# "The Hospital" Nursing Mirror

**Published:** 1897-10-09

**Authors:** 


**"The Hospital" Nursing Mirror.**

Being the Nursing Section of "The Hospital."

[Contributions for this Section of "The Hospital" should be addressed to the Editor, The Hospital, 28 & 29, Southampton Street, Strand, London, W.C., and should have the word "Nursing" plainly written in left-hand top corner of the envelope.]

### News from the Nursing World.

### ST. GEORGE'S MATRONS.

Miss Smedley has not yet taken up work at St. George's, as she has been indisposed. Mrs. Coster, therefore, continues to superintend the nursing as hitherto. This veteran matron has not made any plans for her future; she will visit friends as soon as she is free.

### ROYAL RED CROSS.

Mrs. Ann Ayre Hely and Miss Sarah Terratt have had the decoration of the Royal Red Cross conferred upon them by command of the Queen, in recognition of their services in nursing the sick and wounded.

### A SPOILED CAREER.

The need of more consumption hospital accommodation is painfully enforced by the case of Nurse Norrie at Chelsea. The nurse was a bright, healthy north-country girl, and in attending on the consumption patients in the infirmary unfortunately contracted the disease, which rapidly developed. She received every care, and the progress of the malady was arrested, but she has been obliged to give up her career. She is an orphan, and without means. Her fate has aroused the greatest sympathy in the infirmary and amongst the Guardians, who voted her a gift of £50. The Local Government Board have announced that they consider £20 enough. They have been asked to reconsider the circumstances of the case, and permit the original sum to be given. In event of their still declining to allow it, the gentlemen on the Board of Guardians declare that they will make up the deficiency.

### LADY SANITARY INSPECTORS.

Miss Gray, lady sanitary inspector of Islington, is doing excellent work. During the months of April, May, and June, she made 315 inspections, a large number of visits, detected 74 sanitary defects, and superintended the suppression of 26 nuisances. This record is a strong argument in favour of the Battersea Vestry's wish to appoint a lady inspector for workshops and laundries if the London County Council can be induced to pay half her salary. The Medical Officer for West Ham is urging the appointment of a lady sanitary inspector, who is also a district nurse, "who should devote herself to teaching the poor mothers how rationally to rear their bottle-fed infants, and how to avoid the dangers due to ignorant treatment of measles, whooping cough, and diarrhœa, the special assailants of infants."

### WHAT NEXT!

We reprint a notice which appeared in our enterprising contemporary the *Daily Mail*: "On Thursday Misses Millington and Hurlston will hold a reception at Mitcham Hall, Mitcham, Surrey, in honour of Mrs. Bedford-Fenwick and the nurses who have returned from the late Greco-Turkish war. It will take the form of a garden party with music and tennis, and the returned ladies are expected to appear in the picturesque costumes worn upon the battle-field." All this is most fitting and appropriate. If it is true that nurses braved the bullets of the battle-field, surely they are deserving of all honour, while for those whose strong point lay in their "picturesque costumes" a garden party is quite the proper place.

### RURAL INSTRUCTION IN SANITATION.

It has been well said that the indirect consequences of an action or policy are immeasurably greater than the direct result. The work and influence of district nursing is a happy example of this axiom. The patients nursed, the immediate help given, valuable and far-reaching as they are, can only be an imperfect gauge whereby to measure the incalculable benefit rendered by the spread of the knowledge of the laws of health. Mrs. Clare Goslett, a member of the Sanitary Institute, and lecturer on hygiene to the Queen's Jubilee Nurses, recently gave two lectures at Helston. One, addressed to women only, was on the "Responsibilities of Women in Matters of Health and Sickness"; the other, delivered in the evening to a general audience, and illustrated by lantern slides, was on "How our Bodies are Diseased, and How to Keep Away Disease." Mrs. Goslett's visit and lectures are the preliminary steps towards the establishment of a trained nurse in the district.

### GUARDIANS ON SLEEPING ACCOMMODATION.

The Guardians of Coventry are on the horns of a dilemma. They do not want to spend money, yet the sleeping accommodation for their nurses is so restricted that the ingenuity with which some of them endeavour to evade the report of a committee appointed to inquire into the subject can avail them nothing. At present one nurse sleeps in a box 7 ft. 6 in. by 10 ft., without proper ventilation, boarded off the women's imbecile ward. When a new appointment is made to the staff the nurses are moved about in all directions, like "pieces in a game of chess." Oddly enough, a lady guardian recommends the board to "get along slowly."

### HALF A LOAF.

Campden, near Rhyl, has decided to nurse its sick poor on the Holt-Ockley system. Leaflets explaining the rules, subscriptions, and duties of the attendants (wrongly called nurses) were distributed prior to the meeting, called to consider the advisability of subscribing £30 a year to the Moreton Nursing Association. The Holt-Ockley system gives, as some may be aware, tidy, respectable women a few months' teaching, and turns them out with the title of nurse to wait on the sick of all classes. The 10s. per annum subscribers, for instance, pay 10s. a week for the person's services when they are required, and the 2s. per annum subscribers pay 2s. a week under the same circumstances. It is an undoubted gain for the sick poor to have "a tidy, respectable woman," with a veneer of rudimentary nursing to "do" for them (excepting the family washing) at 2s. a week, but such must of necessity be very inferior nurses. The excuse given for the short training of these women, that country nurses do not require the same skill as the town nurse, is fallacious. The difficulty in obtaining prompt medical attendance in country districts is too obvious to allow it to pass—rather a more highly trained, more experienced nurse is wanted. The Holt-Ockley system is better than none; but when there is the Queen's Nursing Institution to give the best nurse possible, after careful training, why put up with something inferior? Want of money is the only excuse; but surely this cannot be urged for Campden, when the Countess of Gainsborough is the promoter of the scheme, and when a score of influential names are associated with hers in this work.

### ANOTHER ADVANCE.

STUDENTS of midwifery will be glad to hear that an admirable model of a pelvis can be seen at the Midwives' Institute, 12, Buckingham Street, Strand. Midwives desirous of obtaining one should write to the secretary for particulars. If thirty orders are received they can be supplied at about 7s. each.

### SITE OF PROPOSED NURSING HOME, GODALMING.

THE committee of the Godalming Nursing Home are fortunate in having an admirable site offered them by the directors of the Capital and Counties Bank at a nominal price of £50. The plot lies at the corner of Croft Terrace, where it is joined by Butt's Lane, and is fairly high, commanding good views of the neighbourhood. It is surrounded by public spaces and ways on three sides, is easy of access, quiet, and enjoys an abundance of light and air; and, if required, more ground could be obtained at a moderate sum.

### A PRACTICAL EXAMPLE.

THE Bath Guardians have a practical example of the loss occasioned by unskilled labour in the commissariat department. They find that out of 984 lbs. of bread used in the infirmary every week 184 lbs. are wasted, and it is the same with other provisions. The remedy they propose is to have the food sent up in bulk to the infirmary to be cut up and distributed by the nurses as needed. This is only one instance of one problem calling for speedy attention. The workhouse masters are hedged on the one side by the Local Government Board, on the other by the pauper who stands up for his rights, and can demand to see his portion weighed in his presence whether he intends to eat it or not. The Local Government Board are going to institute an inquiry into the dietary and abuse of food in workhouses, at which the masters will probably have an opportunity to state their side of the question.

### THE READING UNION INFIRMARY.

THE new Local Government Board Order is laying bare all the disadvantages under which nursing has hitherto been carried on in our workhouses. The present condition of these institutions makes the following out of the new regulations a matter of much difficulty in many places, and there are numerous indications that an evasion of the order will be attempted. At the Reading Union an appeal is to be made against that part of the regulations which prohibits the employment of a superintendent nurse who has been trained where there is no resident medical officer. At the Reading Union there is no resident medical officer; therefore, no nurse trained there can be promoted to the post of superintendent nurse.

### HOME FOR NURSES, BUSBY.

THE Busby Mansion House, which, it may be remembered, was bought last year by the trustees acting under the will of the late Samuel Johnson Moore, M.D., as a home for overworked and aged Glasgow nurses, has been remodelled for this object, and a handsome laundry added. The gardens and recreation grounds are rapidly being laid out, and it is supposed Miss M'Alpine will undertake the entire management.

### DEATH OF A VETERAN NURSE.

NOT long ago we announced that the Queen had decorated Sister Mary Ellis with the Royal Red Cross for her labours on behalf of the sick in war time. Now we much regret to announce the good sister's death at an advanced age. Sister Mary was one of Florence Nightingale's devoted band of assistants in the Crimean War. It is still unforgotten that the women who tended the sick as pioneers had a very trying and arduous work to perform, and their circumstances were very different to those of the Army Sisters in the present day. It therefore gave much satisfaction that Sister Mary's last days were brightened by an honourable recognition of her services by the Queen. On her return home after the Crimean War Sister Mary Ellis joined the hospital of St. John and St. Elizabeth in Great Ormond Street.

### PENNY-WISE GUARDIANS.

A CURIOUS instance of a desire for economy is shown by the proceedings of the Meriden Board of Guardians. Meriden Workhouse has no trained nurse, but one of the Guardians, at a recent meeting, called to consider the matter of following the direction of the Local Government Board, remarked that he thought the Meriden Board need not trouble to carry out these orders, so long as the officers appointed by the Board gave satisfaction, and there were no reports or complaints of neglect. This proposal, however, was not supported, but it was suggested that if a paid nurse were appointed the Board could dispense with the cook and the assistant matro·. Another suggestion was that the Board need not hurry, as the Local Government Board never hurried.

### ANOTHER IMPOSTOR.

A MAN is just now going round to different nursing and charitable institutions, representing himself as a reporter from the *Daily Graphic*. He asks for a prospectus and any data regarding the institution, for the purpose of writing a notice for the *Daily Graphic*, and then suggests payment in advance for a certain number of the paper containing it, or for copies of slips. He is tall and thin, and appears about 60 years of age.

### SHORT ITEMS.

NURSE PHŒNIX, of the Wigan Workhouse Infirmary, was seized with a fit whilst sitting over the fire. The unfortunate nurse's body was horribly charred before it was discovered.—Miss Mary E. Wadham is bringing the urgent need for trained nurses for the poor in Barrow before the public. Two general nurses and one maternity nurse are required to do the work efficiently, and the estimated cost is £100 per annum for each nurse. The Barrow Ladies' Charity have promised £100 for the first year. No steps will be taken until adequate support is given.

### Practical Aspects of a Nurse's Life.

BY SISTER GRACE.

### VII.—PROBATIONERS IN SURGICAL WARDS (*continued*)—OPERATIONS.

ALMOST all nurses as beginners have a great ambition to attend operations, and imagine that when they have attained to this distinction they will, indeed, be finished nurses. In reality, unless a nurse hopes eventually to become sister of a surgical ward, her attendance at operations is of far less importance than she thinks. The operative work that is of the most practical use to ordinary nurses to watch is that connected with

### MINOR SURGERY IN THE WARDS, where you see the whole thing from beginning to end, help in making all needful preparations, and probably assist the surgeon by holding the limb or part to be operated on; in this way you obtain a thorough knowledge of all the instruments in daily use, and understand the preparations needful to make for opening abscesses, aspirating, ligaturing arteries, &c.

You will have to get everything ready for these minor operations, and as the preparations differ in every case you will find it needs much care and forethought to forget nothing, whereas at operations in the theatre your occupations will be confined to washing sponges or preparing swabs, and studying the backs of the surgeons and students bending over the table. But whether in the theatre or in the wards, study—not how much you can see, but how to make yourself unobtrusively useful.

### AMPUTATIONS

are very frequent in manufacturing districts, where machinery accidents are more or less unavoidable. The treatment of amputation for disease and for accident differs somewhat; in disease the patient is in a more or less bad state of health, run down by previous suffering, and requiring much general nursing besides that necessitated by the operation. You will, however, be interested to see how frequently the general health rapidly improves after the amputation of diseased limbs, especially in such cases as diseased knee joint, when the sufferer has been brought very low. On the other hand, amputations after accidents have their own peculiar dangers; the patient has already sustained a severe shock to the system, and perhaps lost so much blood as to make it a dangerous matter to subject him to further exhaustion. This is the reason why patients who suffer amputation of two limbs at the same time so seldom survive. These cases need very careful watching and tending for some time, but if they recover these repeated assaults upon their strength, they quickly recover, because they have not to battle against the ill effects of a long previous illness. In accident cases you have seldom any opportunity for cleaning and preparing antiseptically the limb to be operated on; for the same reason the bowels cannot be attended to, as is desirable before all operations, and I may here tell you that in accident work the management of the bowels constitutes a serious difficulty; men struck down in the midst of health and strength, and probably eating heartily and somewhat coarsely perhaps, suffer very often from what seems almost a temporary paralysis of the bowels. The sister will explain to you the various methods used to overcome these difficulties. You will, of course, always have hot bottles ready for all operation cases, and a towel and porringer ready by the bed. Some surgeons like a stump raised on a pillow and some not. I like to remove the pillow after a few hours, as it has a tendency to cause the discharge to run back and escape from the dressings at the edge of the bandages. You will find sometimes that there is an inclination to start and twitch in a stump, and a slight, steady pressure with the hand will often relieve this. In future dressings of the stump, as in all surgical work, be sure that your hands are absolutely surgically clean, your nails must be kept short, and never touch a wound without putting your hands into carbolic or whatever lotion is in use. You will receive instruction on the subject of antiseptics, so that it is not necessary for me to enlarge upon it; but in your ardour for science do not forget common-sense, practical cleanliness. There is always a certain amount of danger of secondary hæmorrhage after amputations, and you will be taught how to watch the cases; remember, if it should occur, there is no time to be lost.

### TRACHEOTOMY.

In operations for removal of the tongue or portions of the jaw for cancer, &c., tracheotomy is often combined. These operations are very distressing, and you must do all in your power to cheer the patient under the distressing circumstances in which he finds himself. There is considerable danger of septic pneumonia from the impurity arising from the wound, and you must direct all your energies to keeping the adjacent parts as clean as possible by means of frequent syringing and mopping out with lint fastened on sticks, which will dislodge the tenacious mucus clinging round the mouth and throat. When tracheotomy has been resorted to, it is essential to keep the atmosphere of the room warm and slightly moist by the use of a steam kettle with some disinfecting fluid in it. Keep in mind that a direct opening has been made into the air passages, and therefore the air that reaches them must be artificially tempered. There is in diphtheria a great danger of the patient being suffocated from an accumulation of mucus in the tube, and you will be taught to clean it frequently. See that he does not in a paroxysm of coughing eject the tube; that would be a serious accident in the earlier stages of the disease. When tracheotomy has been employed in cases of excision of tongue, &c., if all goes well the tube is soon removed, as it is in such cases only used for temporary convenience, and the difficulties that arise in the cleaning of the tube are very slight. In diphtheria it is a different matter, and a nurse is usually employed for such cases, who is experienced and reliable. You will find that a solution of soda is a simple and easy help in the cleaning of feathers and tubes used in tracheotomy cases. Be sure that in refilling your steam kettle you always do it with boiling water, so that it may not be temporarily off the boil. The nursing of tracheotomy cases is very interesting, and often taxes the ingenuity of a nurse.

### Appointments.

### MATRONS.

WEST HAM HOSPITAL, STRATFORD.—The newly-appointed Sister of the Westminster and Roberts wards and operating theatre of this institution, Miss Amy Ough, has enjoyed a successful career. She was trained at the hospital for two years, passed first in the examination, and received the prize given by Dr. Grogone. She has been staff nurse for some time of the wards of which she is now made sister.

COTTAGE HOSPITAL, FLEET, HANTS.—Nurse Adah E. Stronghill was appointed Matron at this hospital on September 1st. She was trained at University College Hospital, and subsequently became charge nurse at the Hampstead Infirmary.

### Wants and Workers.

THE Boldmere District Nurse, Wylde Green, Birmingham, would be grateful for any old linen or flannel for a poor woman whose ailment necessitates a yard per day.

### On Preparation for Operation in Private Houses.

By E. STANMORE BISHOP, F.R.C.S.Eng., Hon. Surgeon, Ancoats Hospital, Manchester.

(*Continued from page* 6.)

WE will assume that the operation to be done is an abdominal section. It is a very good plan—it is a method I adopt myself—to make out a list of things as they crop up in going through the operation from start to finish in imagination. Suppose the morning here, and the operation about to begin.

ROOM.—The surgeon has come, and wants a room, properly warm, clean, and well-lighted. This may have been already chosen for you by the surgeon, but, if not, your advice is certain to be asked. It should, above all things, be the lightest you can find. A room at the back with the window facing another block of buildings, with only a couple of yards between, will certainly *not* do if it can be avoided. Choose the largest, lightest room available. Now take out of it all unnecessary things—toilette unnecessaries, chimney-piece ornaments, all loose pieces of carpet, clothes, antimacassars, bed-hangings, anything that will not be required or that can harbour dust. Have the carpet brushed over with damp tea leaves, or, better still, remove the carpet, and have the floor well scrubbed. Open the window widely, and if the room has not been used for a while, or if the weather is cold or damp, have a good fire in to-night, and get the room well aired.

BEDSTEADS.—Next you want a small bedstead, the narrowest there is in the house; if an iron one so much the better. You may have to put up with a large four-poster. Put it in any case so that at first it will take up as little room as possible, and will not encroach on a fair space in front of the window.

OILCLOTH.—This space, for operating, should be covered by a piece of oilcloth, which will protect any carpet or the bare boards from staining by any fluids which may drip from the operating table.

OPERATING TABLE.—Some surgeons provide their own, but generally, I think, you will find it better to arrange one from the materials at hand. Anything like a plank on trestles I think is very objectionable. It has to be brought to the house, and there is something very suggestive of the erection of a scaffold for the next day's execution in the thing. All the neighbours in anything like a populous neighbourhood are excited and agog to watch the process of getting that ghastly board and its supports into the house and up the stairs. If you hint to the surgeon that you can manage very well without it I am sure that he will thank you, and you will have saved your patient one not inconsiderable annoyance. There are very few houses where a stout kitchen table or two are not to be had, and by placing these in the form of a T, with or without bricks under their feet to raise them to a proper height, a very useful and comfortable operating table may be contrived. Have the tops well scrubbed with soap and water. Sometimes one table is sufficient. In any case, get them, if possible, without side flaps—these are always in the way. Above all things, never trust to these flaps; never put them up. If they cannot be removed—and, as a rule, a screw-driver will make short work of them—keep them down. They will drop at the worst possible moment, and nothing will make them safe. In placing the table, unless the Trendelenburg position is wanted, about which I will speak presently, see that it lies rather aslant with reference to the window. The light is wanted to fall upon the abdomen, and if the table is parallel to the wall the surgeon's own body will intercept it. If possible, place the patient's head furthest from the fire for two reasons. First, it is far better and pleasanter for her that her feet should be the warmest; and, second, because the anæsthetist has to stand at the head, and should the operation be prolonged, you will expose him to a severe roasting if this arrangement is reversed. You may chance to obtain a second from him afterwards.

Keep the patient out of the room until the actual time for operation. In the morning the surgeon will have his own preparations to make, and nothing can be more trying than for her to lie in bed and watch the whole process. It would take the last bit of courage out of anyone. For the same reason, see that when she does come in everything like an instrument is covered up. But she will need preparation overnight.

PREPARATION OF PATIENT: BOWELS.—The bowels must be well cleared out, and the method of doing this must depend upon the idiosyncrasy of the patient. Some are very sensitive to purgatives, others require very strongly acting remedies. Find out, if this has not been done beforehand by the surgeon, what is likely to produce one, or, at most, two free actions, and give this at once.

ENEMA.—Follow this up the next morning by a warm enema of soap and water at least an hour before the operation is to be done.

RAZOR, TURPENTINE, SPIRIT.—Shave all hair from any part in the field of operation or near it. Then rub the whole surface well with turpentine, scrubbing it well into the skin, and especially about the navel, with a nail brush; then again scrub with methylated spirit; lastly, with soap and water. The object of these scrubbings is to dissolve out all fatty matter, old perspiration, &c., with its imbedded germs, from the skin, and leave it ready for the action of a compress soaked in mercuric biniodide dissolved in water 1—1,000.

COMPRESS, LINT, MERCURIC BINIODIDE.—This compress is made of three or four folds of lint soaked in the solution, and laid on wet, covered by jaconet and a bandage or towel. This is left on all night.

NAIL BRUSH.—In the morning a lot of old epithelium will be found softened, and easily scrubbed off by the nail brush. The compress, resoaked, should be again applied, and only removed when the patient is anæsthetised.

WINDOW BLIND.—The patient prepared, the room must be still further attended to. All curtains, blinds, &c., which can obstruct the light from above must be removed, but a short window blind will be required for the lower panes, to block the view from trams, buses, opposite houses, &c.

WATERPROOF, BLANKETS, PILLOWS.—On the table you will want a waterproof sheet or a piece of oilcloth, to protect it from stains of blood, &c.; three folded blankets, one placed below the patient, one to go over the chest, and one over the lower limbs, so that the abdomen may be easily uncovered whilst the rest of the body remains warmly clad; two pillows for the head.

HOT AND COLD BOILED WATER; EWERS.—See that there will be a plentiful supply of hot and cold boiled water which is to be cold over night. See that it actually boils for fifteen minutes, and preserve it in two clean covered ewers. See that those ewers are first well scalded out to get rid of dust. See that the water itself is clean; but there is no greater sin against the gospel of asepticity than to place water sterilised by boiling which may have to be used for flushing out the peritoneum in jugs which still contain dust which has been collecting for weeks. I have seen this done, and if there is anything which would justify nursicide, it is that look of apathetic surprise which the offender puts on when such an unjustifiable blunder is pointed out to her. Make arrangements that an equal supply of hot boiled water will be available in similar receptacles in the morning.

Two Small Tables.—Two smaller tables will be wanted, one for the surgeon's instruments, one for the sponges, &c. The first should be so placed that the surgeon can easily reach anything upon it without moving.

Irrigator, Cord.—If the irrigator is likely to be wanted you will want a stout cord, which should be attached to it and thrown over the curtain pole, or a strong nail driven into the window frame at a height of four feet from the table. If obliged to use a nail, use a strong one, and do not drive it into the wall; it will almost certainly give when the weight of water in the irrigator comes to bear upon it. In any case, whether you use a nail or the curtain pole for this purpose, you will want another holdfast for the lower end of the cord, which should be within easy reach, so that the irrigator may be readily raised or lowered at discretion.

### Diet for Typhoid Fever Patients.

In the first stage of typhoid the diet consists of milk and liquid food, so it is not so difficult to arrange for the patient as it is when the convalescing stage is gained. The patient is then ravenously hungry, and yet the return to solid food must be so very gradual. Great care must be taken that anything in the way of gruel and custards, &c., be strained free from lumps, that would cause irritation if swallowed by the patient. In fact, all their food must be specially prepared for them, and be of the lightest and yet most nutritious quality; one return to full ordinary diet might be attended with a fatal result. Beef tea and clear soup are much improved by having a little finest pearl sago boiled in them—mutton and chicken broth, the same, if a little well washed pearl barley be boiled in them, and a spoonful of cream added just before serving.

The following recipes are for various dishes suitable for typhoid cases during convalescence:—

Oyster Broth.—Put six oysters with their liquor into half a pint of mutton broth, quite free of fat; let them simmer gently for ten minutes. Strain the broth through a very fine strainer into a clean stewpan, bring it to the boil, and stir into it half a teaspoonful of arrowroot that has been mixed with a little cold milk; add a pinch of salt and two tablespoonfuls of cream, taking care not to boil the broth after the cream is added. Strain it before serving.

Rice Jelly.—Well wash a quarter of a pound of Carolina rice, put it to boil for one hour in a quart of boiling water, then strain it into a basin or mould; when quite cold, serve it with milk or castor sugar, or it can be melted again and mixed with warm milk and sweetened to taste.

Light Bread.—Rub two ounces of butter into one pound of flour, add a good pinch of salt and one ounce of Cowan's Baking Powder; mix into a light dough with half a pint of milk, put it into two well-greased tins, and bake in a moderate oven for about three-quarters of an hour. This is very nice for bread and butter and sandwiches, taking care to always remove the crust for both.

Chicken Sandwiches.—Cut some thin bread and butter from a light loaf of bread, using good fresh butter. Then spread them with the following mixture: Chop very finely two tablespoonfuls of the white meat of a cooked chicken, pound it in a mortar with a little salt and a dessert-spoonful of thick cream, then rub it through a fine wire sieve, and use as directed.

Tapioca Soufflé.—Soak a tablespoonful of tapioca in cold water, then drain it and boil in half a pint of milk till the tapioca is transparent and the milk rather thick; sweeten with castor sugar and stir in the beaten yolks of two eggs; flavour with a little vanilla, whip two whites of eggs stiffly with a pinch of salt; mix these in gently with the tapioca, pour it into a well-buttered soufflé dish that is surrounded with a band of buttered paper, and bake in a moderate oven for half an hour.

Fillets of Whiting.—Remove the fillets and every bone from a fair sized whiting. Bat out the fillets with a knife dipped in cold water; season them with a little salt, and lay them on a buttered baking tin, pour a tablespoonful of cream over each, and cover the fillets with a piece of buttered paper, stand the tin in another containing hot water, and put them in a very moderate oven for twenty minutes. Dish up on a little dish, and garnish with sprigs of parsley.

Boiled Custard.—Boil half a pint of milk with two ounces of lump sugar and a small piece of lemon peel for five minutes; stir one teaspoonful of cornflour into half a teacupful of cold milk, pour the boiled milk on to it, and let all boil for two minutes. Mix together two yolks of eggs in a basin, pour the hot mixture on them, return to the saucepan and stir over the fire till it thickens, but do not let it boil, strain it, and serve cold in glasses, with a little coloured sugar sprinkled on the top.

Vanilla Cream.—Boil half a pint of milk with two inches of vanilla pod split in two; leave it on the side of the stove to infuse for fifteen minutes, then add to it two ounces of castor sugar and not quite half an ounce of Marshall's finest leaf gelatine; mix this on to two raw yolks of eggs in a basin, return to the saucepan and stir on the fire till it thickens, strain it, and when it begins to set mix into it a quarter of a pint of whipped cream and a few drops of vanilla essence. Set away in a cold place after putting it in a fancy mould. Turn out by dipping the mould in warm water.

Raw Meat Sandwiches.—Cut some thin slices of light bread and butter and spread them with the following mixture, occasionally dipping the knife in hot water: Pass half a pound of fresh lean beef steak twice through the mincing machine; then pound it in a mortar, and rub it through a fine wire sieve; season it with salt, and use as directed.

Devonshire Junket.—Make half a pint of milk blood warm, put it in a glass dish, and stir into it a teaspoonful of castor sugar, and a dessert spoonful of prepared rennet; cover it over and let it set. When cold serve with a little whipped cream on the top that has been sweetened and flavoured with a few drops of vanilla essence.

Barley Water.—Well wash two ounces of pearl barley, put in a jug with five lumps of sugar and a strip of very thinly-cut lemon peel. Pour on to them a pint and a half of boiling water; cover the jug, and when cold stir in the raw white of an egg that has been lightly broken with a fork. Strain the barley water through a fine strainer before using.

Toast Water.—Take the bottom crust of a loaf and toast it thoroughly both sides without burning. Put it in a jug and pour a pint of boiling water on it; when quite cold strain it carefully.

### Minor Appointments.

City of London Infirmary.—Miss Constance Lizette Forey was appointed Charge Nurse of this institution on August 24th. She was trained and subsequently held the position of nurse for three years at St. Luke's Infirmary, Chelsea.

West Ham Hospital, Stratford.—Miss Winifred Massey and Miss Violet Galt have been appointed Staff Nurses at the above hospital. Both were trained for two years in the same.

### Everybody's Opinion.

[Correspondence on all subjects is invited, but we cannot in any way be responsible for the opinions expressed by our correspondents. No communication can be entertained if the name and address of the correspondent is not given, or unless one side of the paper only is written on.]

### THE MAN WITH A BROUGHAM.

"A NURSE" writes: The above case is quite similar to one I read in a weekly paper the week before. A tall, gentlemanly-looking man called at the Nurses' Co-operation, 8, New Cavendish Street, and asked to see the matron. He told her he had been sent by a doctor for a surgical nurse to attend a lady who had met with an accident. The nurse would be wanted for four or six weeks. Having obtained the address of a nurse he drove off and induced the nurse to accompany him. He represented that his master did not like luggage on the carriage, and therefore it had better be sent by train from Victoria Station. After driving about town for some time and borrowing (?) over £2 from the nurse, the man suggested that she should go on to the case while he waited for some dressings, &c., which the doctor had ordered. The address he gave was at Camberwell, and, needless to say, when the nurse arrived the people knew nothing at all about the matter, and the nurse was left to get back as best she could with empty purse and luggage lost. The matter was put in the hands of the police, but evidently the rogue is still at large. Let us hope all private nurses are on their guard and he will get no more victims.

### NURSING AT MAIDSTONE.

MR. ARTHUR B. URMSTON, of Maidstone, writes: As a member of the Special Committee dealing with the typhoid epidemic at Maidstone, I have been requested to write and ask you to contradict in your next issue a statement which has appeared in many papers to the effect that our medical officers and the committee are in need of volunteer nurses for our district and hospital staffs. I am glad to say a regular supply of certificated nurses from well-known institutions has been maintained, and therefore there has been no need to engage independent nurses. A welcome has, of course, been given to a few who wished to come and work among the poorest on their own resources. The Mayor, the medical officers, the members of the committee, and others have received scores of applications for engagement from all parts of the United Kingdom. These, under the pressure of our work, have remained and must remain unanswered; and while thanking them for the expression of their willingness to help us at this time, we trust they will forgive the discourtesy which apparently they have received.

*** In regard to the above letter, it should be noted that the Chief of the Medical Staff Corps has issued a printed circular asking for volunteers to assist in nursing at night, and that it is generally understood that a large number are at present so employed. Of course, if the good people are content with amateur nursing, there is nothing more to be said. But so long as these volunteers are found necessary, it cannot be admitted that nurses are not wanted at Maidstone.—ED. *T. H.*

### COMMON SENSE.

"EXPERIENTIA DOCET" writes: Under the heading "Common Sense," in your issue of last week, you refer to a letter concerning "Nursing as a Calling" in the *Spectator* of September 11th, but will you allow me to point out on the other side of the question why it is a danger that women may take up nursing as they would open a shop or do type-writing? There is no doubt whatever that sentimentalism might be an even worse danger, but its votaries do their harm almost entirely outside, not inside, the profession, the hard facts of hospital life soon driving the sentimentalists out of the wards. But, on the other hand, the crucial difference between "honest devotion to duty" in type-writing and in nursing is that, while in both it may honourably include keeping oneself off the rates—and no one feels more strongly than I do that the labourer is worthy of his hire—yet "duty" in a nurse should mean much more than earning her daily bread. In dealing with inanimate things, no one suffers from the motive being merely self-preservation; but for dealing with human character as a teacher has to do, with human souls as a missioner woman has to do, with human lives as a nurse has to do, much more is required, surely? Honest devotion to duty is without doubt a *sine quâ non* of a nurse's work, but in dealing with human lives, human character, human souls, does it not mean devotion to the service of others in addition? And surely a lower motive can no more carry a nurse honourably and effectively through the long strain of wearing responsibility, of conscientious attention to detail, of perpetual calls upon her sympathy, of more or less constant fatigue, than the need of a stipend can make a good clergyman or the necessity for fees a good doctor in the sense in which we expect them to be good in our hours of greatest need.

### Notes and Queries.

*Treatment for a Child.*

(10) Will you tell me of a hospital near Clapham where I can take a child who is deformed for consultation?—*E.C.*

The Victoria Hospital for Children, Queen's Road, Chelsea, would probably answer your requirements.

*Short Sight.*

(11) Will short sight prevent me from becoming a nurse?—*M.A.*

It depends on the extent of the defect. You had better ask a doctor to give you a certificate if he can, to the effect that your sight is good enough to perform the duties of a nurse.

*Nursing in South Africa.*

(12) Please tell me to whom must I apply for nursing in South Africa —*re* Lambeth Conference appeals?—*Harrie.*

We believe the Church Missionary Society.

*Works on Surgical Nursing.*

(13) Would you kindly tell me the name of a good book on surgery, including preparation and treatment after various operations?—*Enquirer.*

"Surgical Ward-work and Nursing," by Alexander Miles, is most useful (the Scientific Press, 28 & 29, Southampton Street, Strand). A course of practical articles on this subject is now current in the "Mirror."

*Monthly Nursing.*

(14) Could you inform me what hospital or infirmary would train monthly nurses without payment or fees?—*M. Hooper.*

You cannot be trained without paying fees. One of the district nursing associations might possibly pay your fees for you, in which case you would be bound to nurse for that society for a fixed period. In this case the Secretary, Queen Victoria Jubilee Institute, St. Katherine's Hospital, Regent's Park, would best be able to advise you.

*The Best Training.*

(15) Would you kindly tell me if it would be more advantageous to me afterwards to go for three years' training and pay no premium or to pay a premium and go for one year to a large general hospital?—*B. B.*

In any case, three years' training in a general hospital is necessary to qualify for any important position. In some hospitals a premium gives advantages, and in making inquiries you should satisfy yourself on this point.

*Surgical Appliances on Hire.*

(16) Can you tell me of any place from which various surgical appliances and invalid furniture could be hired. I want to draw up a scale of charges. They will be lent free to cottagers and the artizans. (2) I should be glad to know what is done in other places; and (3) what should be the rule as to the length of time the things should be kept. Some months ago I sent these questions to THE HOSPITAL. They were inserted, but I received no answer.—*Mary Holloway.*

(1) Write to the good instrument makers, such as Messrs. Maw, Son, and Thompson, or Messrs. Bailey and Son, Messrs. Alfred Carter, Holborn Circus, and Messrs. Leversons, New Oxford Street. (2) We believe that it is usual to get such things through friends and by subscription, and keep them at the head-quarters of the association. (3) The necessity of the patient must be the only rule as to the length of time they should be lent. Chronic cases should, of course, obtain their own instruments as soon as possible.

*Permanent Non-resident Appointment.*

(17) Could you kindly assist a nurse to obtain some permanent non-resident appointment? She has the highest references, and is London trained, with experience in private work. She is too poor to advertise, as she has relatives dependent on her, and she has been out of work for four months.—*Nurse.*

We insert Nurse's query, and hope that it may be of service to her. Workhouse appointments are now plentiful, or she might see advertisement column in "Mirror," or join a private institution.

*Broken Engagement.*

(18) I was engaged to attend a lady in May, and remained with her seven weeks, but the confinement had not taken place when I left owing to another engagement. Am I entitled to any fee?—*Monthly Nurse.*

Certainly; if you were with the lady at the time originally engaged for you are, at the very least, entitled to half fees.

*Prison Wardress.*

(19) I am 5 ft. 4 in. in height. Would this be tall enough for a prison wardress? I am now assistant matron in an institution.—*Nurse Jessica.*

If you are exceptionally strong we think you are tall enough. We cannot think that you would like the work of wardress after training as a nurse and holding a good position. The work is both monotonous and unpleasant; but if you decide to try apply to the Commissioner of Police at the Home Office.

*Address Wanted.*

(20) Will you kindly tell me the address of the Metropolitan National Nurses' Institute?—*J. K. S.*

The title of this association is the Metropolitan Nursing Association, and the address is 23, Bloomsbury Square, W.C.

